# Omnichannel Communication to Boost Patient Engagement and Behavioral Change With Digital Health Interventions

**DOI:** 10.2196/41463

**Published:** 2022-11-16

**Authors:** Agata Blasiak, Yoann Sapanel, Dana Leitman, Wei Ying Ng, Raffaele De Nicola, V Vien Lee, Atanas Todorov, Dean Ho

**Affiliations:** 1 The Institute for Digital Medicine Yong Loo Lin School of Medicine National University of Singapore Singapore Singapore; 2 The N.1 Institute for Health National University of Singapore Singapore Singapore; 3 Department of Biomedical Engineering College of Design and Engineering National University of Singapore Singapore Singapore; 4 Department of Pharmacology Yong Loo Lin School of Medicine National University of Singapore Singapore Singapore; 5 Arcondis Pte Ltd Singapore Singapore; 6 The Bia-Echo Asia Centre for Reproductive Longevity and Equality Yong Loo Lin School of Medicine National University of Singapore Singapore Singapore

**Keywords:** digital health intervention, omnichannel engagement, behavioral change, communication channels, personalized engagement, health care, patient care, health care outcome, patient engagement, digital twin, DHI, digital health, eHealth, framework, development

## Abstract

Digital health interventions are being increasingly incorporated into health care workflows to improve the efficiency of patient care. In turn, sustained patient engagement with digital health interventions can maximize their benefits toward health care outcomes. In this viewpoint, we outline a dynamic patient engagement by using various communication channels and the potential use of omnichannel engagement to integrate these channels. We conceptualize a novel patient care journey where multiple web-based and offline communication channels are integrated through a “digital twin.” The principles of implementing omnichannel engagement for digital health interventions and digital twins are also broadly covered. Omnichannel engagement in digital health interventions implies a flexibility for personalization, which can enhance and sustain patient engagement with digital health interventions, and ultimately, patient quality of care and outcomes. We believe that the novel concept of omnichannel engagement in health care can be greatly beneficial to patients and the system once it is successfully realized to its full potential.

## Introduction

Digital health is an emerging field that involves the use of information technology for a range of health-related applications—from general wellness to medical devices [1]. A wide range of digital health technologies across the spectrum of mobile health, wearable devices, telehealth, and personalized medicine solutions is being explored. Digital health interventions (DHIs) are digital tools that help modify an individual’s health behavior through direct interaction. These approaches can potentially improve the quality, accessibility, and affordability of health care worldwide [2]. Moreover, due to the availability and convenience of technology access and its immense potential for development, DHIs successfully implemented in a clinical practice can have the capability of driving patients’ health behavioral change in response to real-time tracking of intervention progress.

The COVID-19 pandemic has accelerated the adoption of digital health technologies on a global scale, with large programs already deployed in clinical practice [3,4]. With rapid digitalization of health care coupled with recent efforts to reimburse DHIs (eg, Digitale Gesundheitsanwendungen, also translated as digital health applications in Germany, the mHealthBELGIUM platform in Belgium, National Health Service Scotland) and the emergence of new digital interaction modalities, for example, Metaverse [5], as potential interfaces for DHIs in the future [6], guidance on how to best improve an individual’s health with DHIs is required, including effective patient engagement strategies.

In this viewpoint study, we aimed to identify, in broad strokes, how DHIs could be potentially integrated into future health care. We focus on patient engagement as the critical component to effectively change the behavior of the patient via DHIs [7]. As summarized in a review by Barello et al [8] on eHealth, patient engagement encompasses adherence to treatments and medications and thus directly impacts all relevant outcomes of health interventions [9]. Among others, treatment adherence has been the most recently discussed as a key explanation for the observed reduction in cardiovascular mortality in the polypill trials [10]. By targeting patient engagement, it is possible to reshape behavioral intentions, motivations, and attitudes, as well as provide direct and continuous feedback on the effect of the changed behavior, which can further reinforce and sustain appropriate change [11,12]. Despite its significant role in digital health tool efficiency, patient engagement in the context of “last-foot delivery” of DHIs has not yet been explored to its full potential.

## Communication Channels for Engagement in DHIs and Behavioral Change

**Figure 1:**
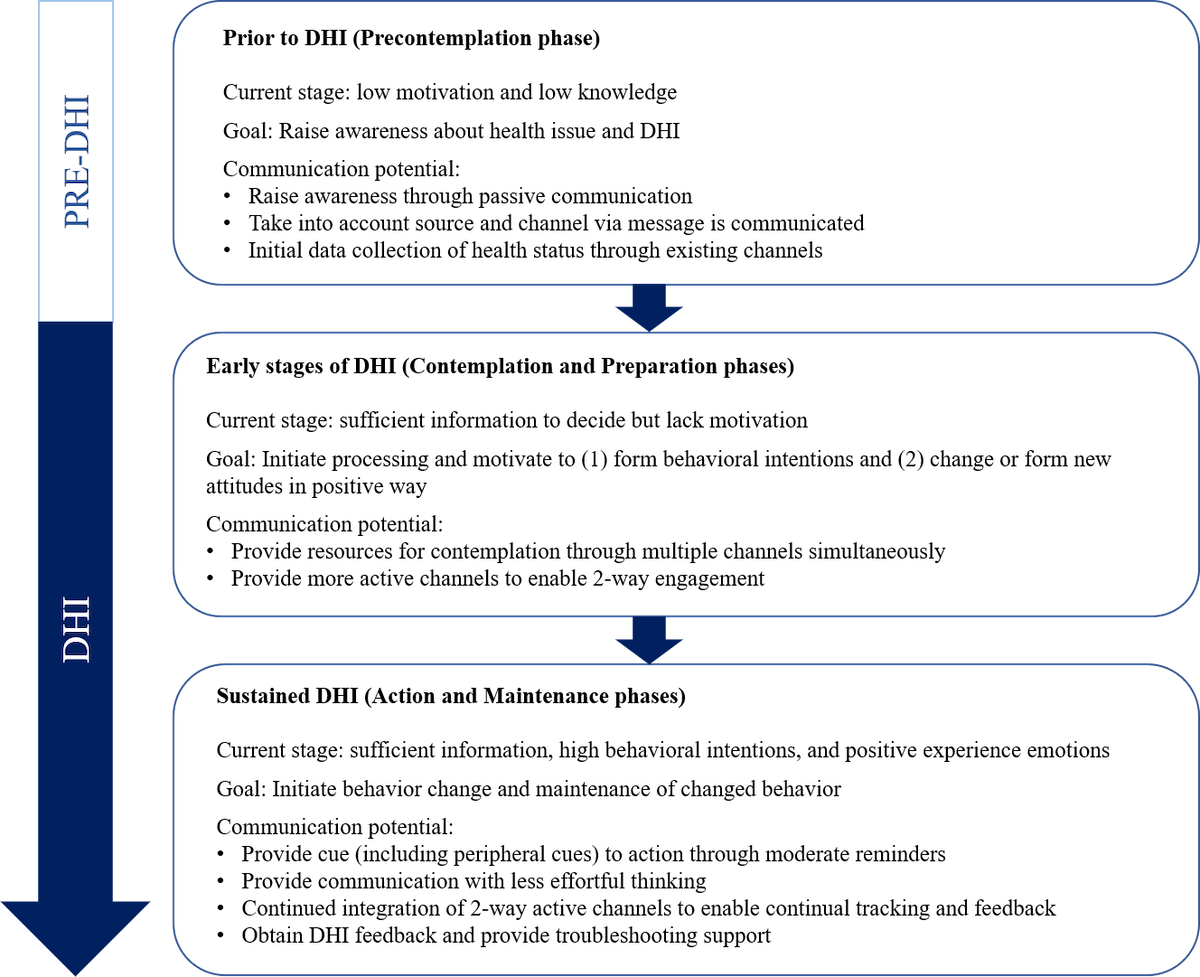
Communication potential of digital health interventions: the potential role of communication toward behavioral change before and during the digital health intervention, as guided by the framework proposed by Manika and Gregory-Smith [12]. DHI: digital health intervention.

Patient engagement is highly interlinked with communication [13] and is a natural extension of the standard clinical patient education [14]. During a DHI, communication represents a dynamic process, which can begin passively (eg, hearing about the intervention through peers or doctors or advertisements), followed by more active interactions during initial and continued usage [15]. According to an integrated conceptual framework for health marketing communications [12], the communication potential and goals vary through different stages of patient interaction with the DHI (Figure 1). Given the variety and adaptability of communication channels [16], deploying the right communication channels and their dynamic adaptation throughout the intervention period can be leveraged as an effective means to build and sustain patient engagement [12]. This consideration precedes the introduction of other epiphenomena of communication, such as gamification [17].

First, patients need to form the motivation for using DHIs by means of prior information and involvement [12]. As such, it is crucial to raise awareness about the health issue and DHIs through physical channels such as health care providers and traditional mass media and virtual channels such as social media. As each patient’s awareness about DHIs is increased, communication channels can evolve to become more personalized and engage patients more actively. During this stage, promoting the motivation to change and forming behavioral intentions and new positive attitudes are essential [12]. Increasing autonomous motivation has been shown to lead to positive health behavioral changes [18], and simultaneous use of multiple communication channels has the potential to employ a variety of motivation and behavior change techniques [19]. Eventually, as patients progress through the DHI process with high motivation and engagement, the goals of communication channels should shift toward initiating and maintaining changed behavior through less effortful thinking. At this stage, continued cue to action through moderate reminders and the use of peripheral cues such as recommendations from doctors and discussion with other patients can be beneficial [12]. Different communication channels can serve different roles and have varying effectiveness on engagement (see Dahl et al [20] for the list of omnichannel touchpoints and perceived effectiveness). Table 1 explores the potential application of communication channels and probable patient adoption considerations as inferred from existing literature on implementing these channels into DHIs. Of note, given the fast-paced nature of technology development, inventories of communication channels will continue to develop as new channels are established and older channels are phased out. With a diverse list of communication possibilities, patient engagement will remain dynamic as informed by the users’ changing communication preferences, the stage of their DHI journey, and the information collected through the longitudinal intervention monitoring.

For optimal personalization and seamless integration, the DHI needs to adapt to the user communication preferences, which vary with demographics, psychographics, and the specific health condition [35,36]. Broadly, young adults prefer solutions that blend in with their current usage—accessible through existing hardware and software, have a familiar syntax (eg, use of emoticons), and are aligned with their existing habits [37]. Older adults, often with limited digital skills, also prefer familiar or easy-to-use communication channels for DHIs [35,36]. Despite the stated importance of a seamless and personalized experience in health care across physical and virtual channels [38], many of the current DHIs are unable to integrate seamlessly with each other and the rest of the health care system to personalize and improve engagement. The lack of integration of DHIs in the patient journey can exacerbate the existing health care fragmentation, which is particularly evident in the gaps in the continuity of care between the hospital and outpatient treatments. The effect of this discontinuity in user experience, among others, shows in the attrition rates with digital health apps. In one of many examples, the usage analysis of 57 mental health apps showed that the percentage of users who opened the app dropped from 69.4% to 3.9% within the first 15 days [39], potentially rendering them ineffective. This rapid disengagement is a particularly detrimental issue for DHIs that target chronic diseases, where continuity of care is crucial.

**Table 1:** Examples of possible communication channels, their potential applications, and some of the patient adoption considerations. Any of such lists should be dynamically updated to match the evolving changes in the individual’s digital presence and preferences.

Patient/caregiver interface	Examples of potential applications	Patient adoption considerations	
		Preintervention	Intervention and postintervention
In-person visit	Consultation with a physician, a rehabilitation session in a center, monitoring and drug titration with specialized nurses	Synchronous communication, requires initiative and time on the patient’s part	Active communication channel
Email	Email consultations, communication of test results, e-referral	Asynchronous communication, both convenient and nondisruptive in the day-to-day lives of patients	Passive communication channel
Video consultation	Teleconsultation, e-prescription, e-referral	Synchronous communication, initial adoption can be affected by ease of use and perceived usefulness [21]	Active communication channel can be used to replace in-person visits for higher engagement but patient satisfaction is essential for continued usage [22]. Common issues include problems with internet connectivity and accessibility [23].
Mobile apps	Wellness apps, screening and monitoring solutions, or digital therapeutic interventions, with virtual reality–based therapy, personalized nudges, and real-world evidence collection	Initial adoption can be affected by ease of usage and user interface. Involvement of health care providers and public health authorities can benefit adoption [24].	Ease of use in long term enables disease monitoring and symptom tracking. Usage can be sustained through generating electronic satisfaction [25].
Digital social environment	Social media and metaverse that can be used for educating, supporting, and influencing behavior change as well as individualized lifestyle improvement	Adoption might be affected by digital divide and privacy concerns.	Enables patients’ engagement with the ability to share, comment on, and react but also social norms intervention, especially for the young adults [26]
Web-based platforms	Web-based learning/educational platforms such as internet-based diabetes management platform or digital patient-reported outcome measure and electronic patient-reported outcome platforms	Adoption determinants include reliability of provider, level of awareness toward web-based platform, and accessibility [27].	Enables self-management. Continued engagement relies on confirmation of user’s initial expectations and perceived usefulness [28].
Messaging solutions	SMS-based communication, chatbot, virtual health assistant, messaging apps for just-in-time interventions, triaging	Familiar platform and increased ease of usage to encourage adoption. Limited with regards to nature and amount of information shared. Narrative persuasion through first-person point of view can impact persuasion of health messages [29].	Enables reminders for chronic disease management. Limited in terms of clinical data sharing—quality of responsiveness and variability can help improve conversation quality [30].
Safe messaging apps	Direct messaging to health care providers, appointment booking, patient reminders, data sharing between health care providers, and patient data storage	Adoption is influenced by convenience and the integration of safe messaging apps to electronic health record systems [31]	Continued usage relies on patient’s preference for enhanced security features and ease of use [32]
Voice-based assistant	Smart home and mobile-based digital assistant that enable proactive reminders, remote care access, and the ability to capture patients’ responses to artificial intelligence–powered voice biomarkers could also be used to screen for and diagnose a wide range of diseases as well as triaging.	The relative simplicity lowers traditional technological barriers and provides the benefits of hands-free and eyes-free engagement mode. The disadvantages are that some patients may have difficulty in formulating a structured sentence for a command as well as may have privacy concerns associated with having a voice assistant “always-on” [33].	Potential for passive symptom tracking and just-in-time interventions
Wearables and sensors	Global positioning system tracking devices, Bluetooth beacon technology, body vitals sensors	Initial adoption can be affected by novelty of wearable technology—for example, design aesthetic is a prominent factor when influencing behavioral intention [34]	Enables passive symptom tracking and generates long-term data for feedback and analysis. Continued perceived usefulness and positive attitude need to be maintained [34].

## Omnichannel Engagement for DHI-Led Behavioral Change

Encouraged by its success in retail [40], we identified omnichannel engagement (OCE) as a highly promising and untapped approach for DHI patient engagement. OCE is a strategy to integrate and interconnect multiple communication channels in a synchronized operating model, which leverages data and digital tools to deliver a seamless, consistent, and personalized experience for the user [41]. The key features of OCE are (1) integrated and interconnected communication using multiple available channels, (2) synchronized/coordinated activity, (3) personalized and consistent experience for the user, and (4) continuous improvement via data and digital tools.

The correct management of the following 6 enablers is crucial for achieving a successful OCE: people, platform, service and content, channels, legal framework, and data and measurement (Table 2). Several consumer technology companies have maximized their profits by executing long-term omnichannel strategies based on a thorough management of these enablers. The consumer technology omnichannel approach is therefore being considered the state-of-the-art and a reference for other industries [42]. Regarding the patient digital behavior, studies show that individuals who seek and are capable of utilizing eHealth information are also more open to use health services provided digitally [43]. Accordingly, health care companies and public sector utilize new ways of doing marketing, where medical science liaisons—services for patients and health care professionals—use increasingly more digital means besides the traditional in-person communication channel [44]. For example, social media posts on Twitter and Facebook have been used by the largest hospital chain in Turkey [45] as well as by the Portuguese national health service to improve health literacy on disease prevention and well-being together with other nondigital channels [46]. The electronic word-of-mouth–based strategy on social media for pharma marketing on over-the-counter medications has also been explored [47]. It is not surprising that the health care market has been targeted in recent years by the consumer technology companies familiar with OCE, which have introduced fitness apps, wearables, and activity trackers [48].

We stipulate that OCE can play a significant role as an integral element of DHIs and not only as an adjacent marketing stream. We envision a patient journey where DHIs are a crucial part of a stand-alone or auxiliary treatment and behavioral change and leverage OCE to boost its efficiency. In our vision, DHIs are distributed into several services (eg, dedicated disease service, tracking service), which are integrated with various communication channels as well as wearables/sensors and the digital presence of the patient. The information flow is bidirectional—the patient receives the digital intervention through OCE and provides information for the DHI operations. All available relevant data, for example, medical data, health data, connections, digital preferences, and data for health can be centrally collected and integrated to form a digital twin [49,50]. Digital twin is a digital representation of the individual whose data it is based on. It can offer insights about that individual, which, in turn, facilitate the design and monitoring of an intervention. The digital twin in our vision may not need to encompass all available data but merely may need access to multiple sources such as the personal health record to gain sufficient insights to be actionable. For example, to understand the phenotypic response and to recommend an optimal pharmacological dosage with sufficient safety and accuracy, only 3-6 data pairs may be necessary [51,52]. Artificial intelligence could prove crucial in managing and extracting relevant features from the multidimensional, noisy, and incomplete data as well as in following up with a variety of appropriate adaptations and actions [53,54]. It will be required to find the right balance of complexity and size of the data model versus the quality and rapid generation of actionable insights, according to the general premise of efficient use of artificial intelligence in health care [55].

OCE serves the purpose of linking the patient to a network of health services through various integrated communication channels revolving around the patient’s health-seeking experience. In already existing examples, instant messaging, be it stand-alone (eg, WhatsApp) or platforms linked to social media such (eg, Facebook Messenger), can be used for triaging in-person and web-based consultations [56]. Wearables enable long-term symptom management, and emails and teleconsultations provide a seamless and convenient method for follow-ups and communication of clinical data. These services can be combined with the aligned nudges for lifestyle and nutritional choices delivered according to the patient’s digital presence, for example, specific food suggestions when shopping online.

OCE also creates opportunities for obtaining additional insights. An analysis of the interconnection between communication channels may allow for analysis of the network of communication and support, which can be a powerful ally for behavioral change. In 1 example, obesity is known to spread through social ties through a diffusion of unhealthy behaviors [57], including physical inactivity [58]. Accordingly, social connections become informal channels of information with real impact on health decisions for self and others, as observed in parenting [59]. Indeed, rapid diffusion of information has been demonstrated within social networks [60]. Such insights could lead to broader understanding of one’s behavior motivators and their environment to launch a precise DHI and form beneficial habits.

**Table 2:** Key enablers for effective omnichannel strategies.

Enablers	Definition
People	Clear roles and responsibilities around governance, omnichannel engagement expertise, skills, and know-how
Platforms	Foundational technology stack
Services and content	Engagement plans of defined customer-centric services, with modular and reusable content
Channels	Integrated operations across all customer-facing channels
Legal framework	Multimedia consent, cookie and privacy policies, medical and regulatory review policies
Data and measurement	Customer identifier, data management strategies for optimal analytics and insights

## Discussion

### Supporting the Patient Journey With DHIs and OCE

**Figure 2:**
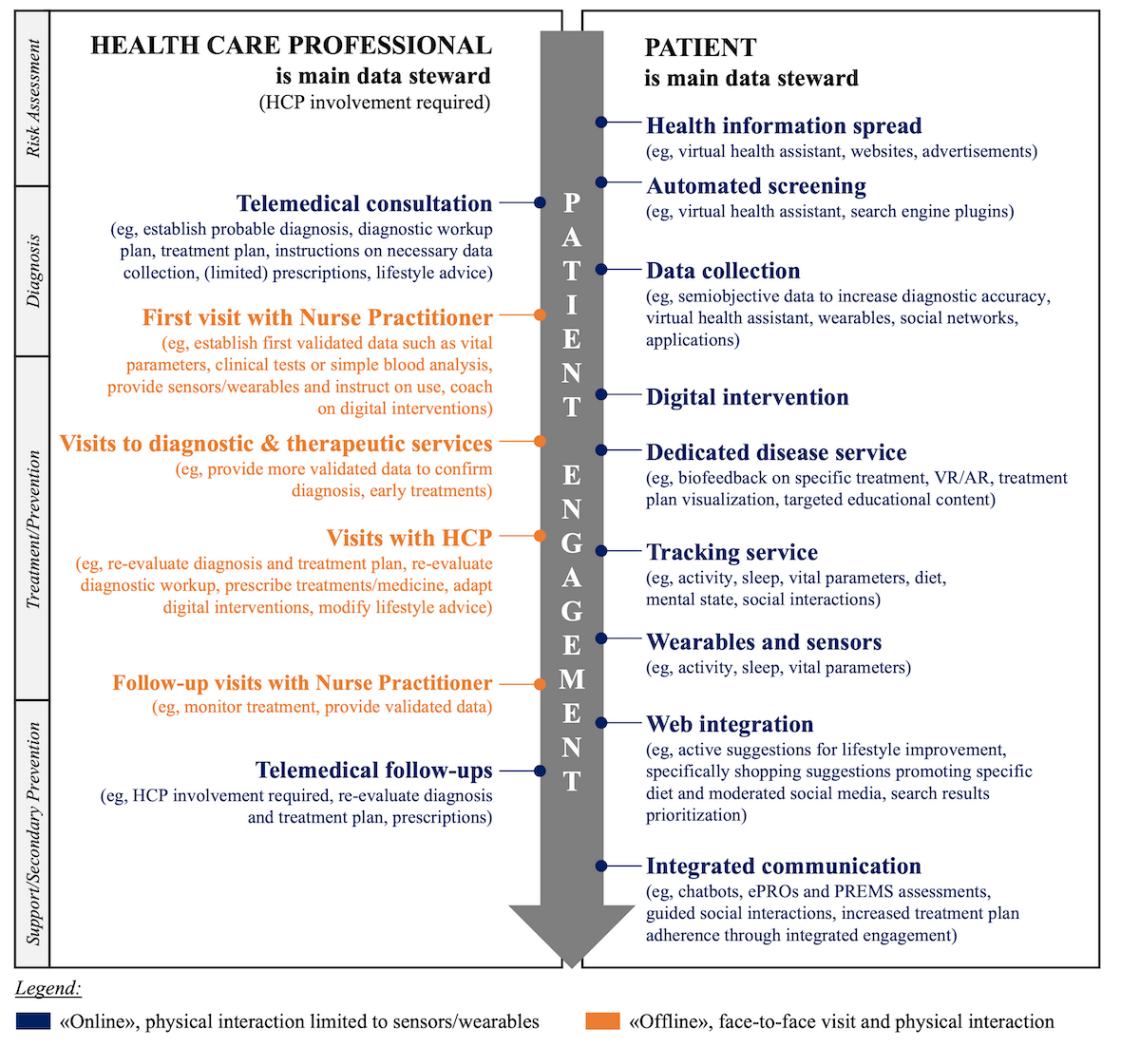
Patient journey with a digital health intervention through omnichannel engagement. The section on the right side of the arrow presents selected communication channels with direct interface to the patient, where patient is the main data steward. The section on the left side of the arrow provides selected communication channels, where the patient interacts with health care professionals who are the main data stewards. The engagement approach with patients is constantly adapted depending on the preferred patient channels and based on the approach that results in an effective behavioral change response. Health care professionals should therefore be channel-agnostic. The data are owned by the patient with stewardship granted to the main contributors (where appropriate). Data actively create a digital twin that enables actionable health predictions and sustained behavioral change. AR: augmented reality; ePRO: electronic patient-reported outcome; HCP: health care professional; PREM: patient-reported experience measure; VR: virtual reality.

In this viewpoint paper, we outline how DHIs supported by OCE may become an impactful health intervention. Below, we describe how a patient journey benefiting from such a tool could look like (Figure 2). At the beginning of the patient journey, individuals at risk of a disease receive broadly targeted medical information through passive communication, with which they can decide to follow up. Interested individuals are supported by an automatic screening procedure in deciding whether contact with a health care professional is necessary. If this is the case, the initial contact is a telemedicine consultation. This allows for a low-cost and rapid way of establishing an initial differential diagnosis, diagnostic workup, and treatment plan. The patient can be instructed on the necessary data to be collected, for example, symptom diaries, wearables, or other tracking platforms, to improve the positive predictive value of the diagnostic procedures. Once sufficient data have been collected, an in-person visit with a health care practitioner follows, during which more clinical data are gathered and the treatment plan is adapted accordingly. Ideally, necessary additional wearables and the correct instructions for new or already used ones are provided at this appointment, together with a recommendation/prescription of an appropriate DHI. Following this are 2 parallel tracks: while the DHI is applied to the patient to change behavior and monitor progress/treatment compliance, the patient also follows up on visits to diagnostic services (eg, imaging services), therapeutic services (eg, physical therapy, nutritional specialist), visits to the doctor’s office, and further visits to the health care practitioner as necessary. We envision that, thanks to the adaptive engagement with DHIs through OCE, patient adherence to both web-based and offline components is high, leading to a rapid and sustained shift in behavior and ultimately better outcomes while reducing the most resource-intense engagements, that is, face-to-face visits. Once treatment is completed, DHIs continue to support the habituation of behavior and thus provide secondary prevention. In patients with chronic diseases, telemedical visits continue as necessary for treatment plan adaptation, while DHIs provide a much closer view of the disease state and offer an asynchronous way for the health care practitioner to stay up-to-date on the patient status.

To allow the flexibility necessary for this approach, it is crucial that health care professionals are agnostic about the effective engagement channel and that their primary systems used to document clinical data support the integration with the patient’s digital twin. Moreover, health care professionals may be required to assess individuals who will most likely need assistance with the familiarization and use of DHIs [61]. Although patients are increasingly open to incorporating technology as part of their care delivery [62], continual efforts through personalization and understanding the resources and capabilities of targeted patient populations should be made to promote greater technology access and literacy. As digital health technologies continue to augment health care professionals’ roles, new skills may become necessary and a new role of a digital health specialist may emerge as a primary health care provider in the future.

### Challenges to Implementing DHIs With OCE

The presented approach, incorporating a digital twin of the patient as well as OCE, needs to align with the collectively accepted principles and values such as “privacy, equity, fairness, patient safety, and patient autonomy over health-care decisions” [63]. Although DHIs may aim to pragmatically increase patient autonomy, the data they use and generate can be seen as a commercial asset, giving rise to risks such as the breach of data security or privacy as well as data governance issues. Adding to this complexity, DHIs operating in multiple countries will need to comply with local, national, regional, and international standards.

Data privacy need to be ensured while using available communication channels and tracking patient preferences and managing the intervention via the digital twin. DHIs have to comply with the prevailing regulations, for example, the general data protection regulation (GDPR) in the European Union as well as with further specifications of each of the European Union member states where a DHI is deployed [64]. The requirements pertain to the physical location of data, the way in which patient consent is gathered, and the necessity for consent before sharing data with specific third parties such as insurers. A specific example in the context of DHIs with OCE is the integration and use of WhatsApp [31]. WhatsApp is a popular messaging app with great potential as a seamless DHI communication channel; however, it fails to comply with GDPR regulations, as messages are stored in servers outside the European Union. In fact, a vast majority of health apps fall outside the remit of the regulations in most nations [65]. A range of secure messaging apps with increased data privacy are being developed and marketed [66-70]. However, their compliance with GDPR varies greatly and questions remain on how they can ensure a seamless experience and sustained use to be potentially adapted for future integration with DHIs.

Patients should be empowered with increased information and awareness of their own health care journey as authorities establish the balance between data protection and data exchange and as new governance models arise. Patients should be given the opportunity to participate actively in their own health decisions and treatment by fully utilizing their health data rights. Among others, an open question remains on the mechanisms to guarantee patient-controlled access to their own data and broadly speaking to the digital twin. The digital twin represents an actionable collection of information designed specifically to be able to “connect the dots” and both predict, monitor, and influence a patient’s behavior. Conceptually, new decentralized technologies such as blockchain may support the control over access and secure information flow in the digital twin [71,72]; however, those are not yet broadly adopted, and beyond technical challenges, a regulatory shift and user education are required to minimize the risk of the digital twin being used as a means to influence the autonomy of the health care decisions.

Some regulatory bodies have issued laws attempting to realize publicly controlled digital twin–like patient profiles (eg, the National Electronic Patient Record in Germany, the National Electronic Health Record in Singapore, the Electronic Patient Dossier in Switzerland). The key benefit of such data exchange systems is to overcome the challenges encountered with historical system interoperability issues and to enable data exchange between health care professionals. While demonstrating significant developments for integrated patient profiles, those efforts have not yet reached a comprehensive patient-specific, actionable digital twin, and the vision of the patient journey illustrated in Figure 2 is not yet facilitated.

### Conclusions

In this viewpoint paper, we have outlined the principles and challenges of DHIs combined with patient engagement through OCE. The evidence stemming from trials and real-world data will verify the true value of such an approach. One strategy to rapidly pilot potential OCE for DHIs could be the combination of a wellness/lifestyle intervention with a health care intervention. This could provide initial evidence for increased patient engagement while testing the concept of continuous improvement with data generated through the engagement. Further integration, for example, with a personal health record could then be added in the second phase. However, we do not see this as a bypass for adhering to the appropriate health care and data privacy regulations. Recently, there have been worrisome reports of the breaches of data privacy and data security by wellness mobile apps [73-75]. In addition to data breaches, other concerns include the aggregation of multisource data and generating digital twins without appropriate access control for the individual represented by the digital twin.

The global strategy on digital health 2020-2025 states that digital health is a means of promoting equitable, affordable, and universal access to health for all, including the special needs of groups that are vulnerable in the context of digital health [76]. To truly fulfil that role, DHIs need proof of effectiveness, accessibility, feasibility, sustainable resource use, and the observation of equity and rights [2]. The extent of success of DHIs in changing health outcomes depends on the level of patient engagement during the intervention and subsequent sustained change of the intended health behavior [77]. Capitalizing on the adaptability of OCE, there is potential for personalizing the user experience to each patient and the flexibility to adapt to the needs and regulations of different geographical regions. Unanswered questions remain with regards to the emergence of new communication channels and behaviors in a digital space as well as adoption of health care models and health care professionals’ roles. In addition, careful management of the commercial versus health care interests in the integration of the communication channels and the digital twin is required. However, we believe that DHIs focused on patient engagement through a dynamic adaptable OCE approach and built on a well-formed compliant digital twin have the potential to disrupt unsustainable health care systems and greatly improve the health of many individuals.

## Abbreviations

DHI: digital health intervention
GDPR: general data protection regulation
OCE: omnichannel engagement
